# Supplementary material for: Learning curve and surgical outcome of robotic assisted colorectal surgery with ERAS program
Source: Sci Rep. 2022 Nov 29;12:20566. doi: 10.1038/s41598-022-24665-w (PMC9709162; doi:10.1038/s41598-022-24665-w)
Supplement: Supplementary file 1 — Supplementary Information 1. [file 41598_2022_24665_MOESM1_ESM.zip › LSC Raw data-Ñ[▒K/086/H_ñw╝╨░O▒Kñσ.pdf]

正式摘要(電子簽章：000000195992918)

W83 / W83 ☐ 英文標題(注意:無英文姓名-請補病人基本檔!) ☐【讀取】憑證

臺中榮民總醫院

出院病歷摘要

轉入號 : 轉出號 :

[redacted]

(8)流水編號	
(9)入院日期	109/07/21 (2020) 直腸外科 W83 - 051
(10)轉科日期	
(11)出院日期	109/08/01 (2020) 住院天數計 11 日

診斷

- 入院:1. Volvulus complicated with bowel obstruction with transitional zone at recto-sigmoid level disclosed by CT,
  -s/p urgent loop colostomy on 2020/6/26
  2. Systemic lupus erythematosus since 1998/2, inducing Lupus cystitis with reflux and bilateral recurrent pyelonephritis s/p bilateral PCN insertion since 1999/4/3(renew on 06/10) and History of lupus abdmoinal vasculitis in 2013/06, under methasone 0.5#QD, plaquenil 2#QD, Imuran 0.5#QD
  - under Methasone 2.5mg IV QD, hold Imuran temporary
  3. Sleep disorder
  4. Bronchial asthma, moderate persistent
- 出院:1. Volvulus complicated with bowel obstruction with transitional zone at recto-sigmoid level disclosed by CT,
  -s/p urgent loop colostomy on 2020/6/26, s/p laparoscopic left hemicolectomy on 2020/07/22
  2. Systemic lupus erythematosus since 1998/2, inducing Lupus cystitis with reflux and bilateral recurrent pyelonephritis s/p bilateral PCN insertion since 1999/4/3(renew on 06/10) and History of lupus abdmoinal vasculitis in 2013/06, under methasone 0.5#QD, plaquenil 2#QD, Imuran 0.5#QD
  - under Methasone 2.5mg IV QD, hold Imuran temporary
  3. Sleep disorder
  4. Bronchial asthma, moderate persistent

主訴

- for scheduled operation

病史

- This 44 years old female patient
  1. Volvulus complicated with bowel obstruction with transitional zone at recto-sigmoid level disclosed by CT,
  -s/p urgent loop colostomy on 2020/6/26
  2. Systemic lupus erythematosus since 1998/2, inducing Lupus cystitis with reflux and bilateral recurrent pyelonephritis s/p bilateral PCN insertion since 1999/4/3(renew on 06/10) and History of lupus abdmoinal vasculitis in 2013/06, under methasone 0.5#QD, plaquenil 2#QD, Imuran 0.5#QD
  - under Methasone 2.5mg IV QD, hold Imuran temporary
  3. Sleep disorder
  4. Bronchial asthma, moderate persistent
  She was just discharged on 7/4. During last admission, loop colostomy was done due to bowel obstruction. She recieved OPD follow-up. This time, under the relatively stable nutritient status, she was admitted for scheduled 2nd stage operation of laparoscopic anterior resection and anastomosis.

體檢發現

- 1070518 dsDNA Ab 8.4 normal CRP 0.095 normal Procalcitonin 0.027
  ....
  1080812 0.034 0.90 13340 8.7 307K 9.3 89.3/27.2 midlly drop of completement
  1080911 0.89 8320 9.1 376K 3.6 84.1/24.6
  1080916 0.113 1.03 12230 9.0 394K 4.7 84.6/31.6

- 1081015 0.072 0.86 11910 8.8 425K 7.2 82.1/25.8
  1081216 0.092 0.92 12250 8.9 416k

Plt 9908 445K->100Feb 371K -394K ->>103/4 346K

allergen specific IgE to DP DF Cockroach & cat/dog dander cadida all negative, further check for inhaled allergenindicated

UP/Cr 4.55 Cr 0.9 GPT 19->100Feb UP/Cr 3.57, serum CREAT. 0.9 mg/dl..102/1 UP/Cr 4.56g (0.7-1.4)

100/9 PCN culture: E.coli, Pseudomonas aeruginosa

106/11 GPT 11 Cr 0.90 WBC 7360 Hb 9.6 plt 433K dsDNA Ab 7.8 C3/C4 80.7/24.7 UP/Cr 1.80g
106/12 CRP dsDNA Ab 9.8
107/1 dsDNA Ab 8.7 C3/C4 91.3/26.6 1070105 ESR 37 hsCRP 0.081
107/3 just with chanmegs of PCN, hgsCRP 0.807 dsDNA Ab 10.1 C3/C4 82.8 23.9
10705 dsDNA Ab 10.5 C3/C4 79.3/24.3 WBC 13100 Hgb 9.6 512k hsCRP 0.190

- General appearance :
  - easy looking , conscious clear , without cardiopulmonary distress
  - Vital signs自動帶入date 體溫(BT) 脈搏(P) 呼吸(R) 血壓(BP) 身高(BH) 體重(BW) BMI
    20200721 36.9℃ 98次/分 18次/分 132/69mmHg 164.6cm 30.9kg 11.4
  - Vital signs: B.P. 132/69 mmHg , P.R. 98 /min , R.R. 18 /min , B.T. 36.9 ℃
- HEENT :
  - Eyes: no anemic conjunctiva, no icteric sclera, normal EOM
  - ENT: no otorrhea, no rhinorrhea, no erythematous mucosa, no injected tonsil, no deformity
- Neck: supple, freely movable, trachea midline,no deformity, no engorged jugular vein , no palpable thyroid or lymph node enlargement
- Chest:
  - Inspection: Grossly normal, symmetric and fully expansion, no accessory muscle used, no operation scar, no spider angiomata, no gynecomastia
  - Palpation: no palpable mass or nodule, no subcutaneous emphysema, no tenderness
  - Ausculation: clear breathing sound
- Heart:
  - Percussion: normal heart size
  - Ausculation: regular heart beat, normal S1, S2, no gallop, no murmur
- Abdomen:
  - Inspection: in
    - flat contour, symetrically, no scar, no stria, no dilated veins, no rashes
  - Ausculation: normoactive bowel sound, no abdominal bruits
  - palpation: soft, no muscle guarding or rigidity, no local tenderness, no rebounding pain, no palpable mass, no palpable pulsation
  - Percussion: no shifting dulllness, no hepatomegaly, no splenomagaly
-

Peripheral pulsation :	CA	BA	RA	FA	PA	PTA	DPA
Right side :	++	++	++	++	++	++	++/+++
Left side :	++	++	++	++	++	++	++/+++

- Genito-anus: no tenderness or palpable mass lesion or rectal shelf, normal external genitalia, normal anal tone, brown stool

CRS CONDITION

-
  - Lung

手術日期及方法

- 手術日期及方法20200722 Laparoscopic Anterior resection and anastomosis-benign

住院治療經過

- During hospitalization, her clinical condition was stable. The operation was done on 07/22. Fever up to 39'c was noted on 7/24, but infection was less likely. No more fever episode was noted after 7/24. She was discharged after patient education. We will folloe up this patient at our OPD.

合併症

一般檢查

- 累積報告：

DATE	TIME	ALB	TP	BIL.T	BIL.D	ALKP	AST	ALT	LDH	CHOL	TG	HDL-C	LDL	UA	NH3	AMY
1090624	1207			0.3				21								
1090625	0147			0.26				17								
1090626	0909	2.8					35	13								73
1090629	0914	2.6		0.24			29	16	256							
1090707	1646							30								
1090715	1018	3.6		0.37	0.09	104	31	29	276							
1090722	1650	3.0		0.3	0.1	62	26	20								
1090728	0918						16	3								

DATE	TIME	WBC	RBC	HGB	HCT	MCV	PLT	BLST	NEUT	BAND	SEG	LYM	MON	EOS	BAS
1090624	1206	16830		10.0			407		75.4			19.1	5.0	0.1	0.4
1090625	0146	17240		8.9			435		83.5			11.2	4.9	0.1	0.3
1090626	0903	14500		8.3			428		90.0			5.9	4.0	0.0	0.1
1090627	0853			8.2											
1090629	0906	13430		7.8			306		88.1			6.1	5.7	0.0	0.1
1090630	0909	16910	3.94	10.1	31.4	79.7			91.6			3.8	4.4	0.0	0.2
1090707	1646	13020		11.4		83.5	487		92.1			5.7	1.9	0.0	0.3
1090715	1018	12750	4.69	11.8	38.6	82.3	470		68.6			24.5	6.2	0.2	0.5
1090722	1650	9130	3.66	9.7	30.0	82.0	304		93.9			4.8	1.1	0.0	0.2
1090724	0925	14840		9.5					86.3			8.8	4.6	0.1	0.2
1090728	0900	12080	3.65	9.6	29.8	81.6	308		82.6			8.5	8.8	0.0	0.1

DATE	TIME	NA	K	CL	CA	BUN	CREAT	Mg	P	CRP	ESR	PCT	LIPASE	LACTATE
1090624	1207	139	3.2			20	1.04			1.730			99	
1090625	0147	139	2.5	108	7.5	15	0.95	1.6	4.2					5.1
1090626	0909	142	3.5		7.8	11	0.73	2.0						
1090627	0859											0.315		
1090629	0903										64			
1090629	0914	137	3.4	105	7.5	12	0.66	1.4	2.5					
1090629	0950													6.7
1090707	1646						0.98			0.867		0.073		
1090715	1018	137	4.3		8.9	12	0.98							
1090722	1650	141	3.6	111	8.0	20	0.99							
1090724	0853	141	3.3			23	0.97			10.148				
1090728	0918	137	3.6	111	7.5	21	0.65							

DATE	TIME	GLUCOSE	PAB	TRF	ZN	CU	KETONE	CK	CKMB	PT-P	PT-C	APTT-P	APTT-C	TRO-T
1090625	0146									10.1	10.3	24.9		
1090629	0914							153						
1090715	1018	65								9.8	10.3	26.8		
1090722	1650	91												

DATE	TIME	SP-GR	PH	PROT	GLUC	KETON	BILI	UROB	OB	NITR	LEU	RBC	WBC	EPCEL
1090624	1143	1.015	6.0	2+	Negative	Negative	Negative	0.2	1+	Negative	3+	5-10	3+	20-30
1090702	2046	1.007	6.5	2+	Negative	Negative	Negative	0.2	1+	Negative	2+	2-5	5-10	0-1
1090702	2046	1.005	6.5	1+	Negative	Negative	Negative	0.2	2+	Negative	2+	2-5	+	2-4
1090707	1629	1.005	6.5	1+	Negative	Negative	Negative	0.2	1+	Positive	3+	5-10	+	4-6

DATE	TIME	STOOL-OB	FAT	RBC	WBC	PARA
1090626	2129			0 - 2	-	Not Found

DATE	TIME	PH	PCO2	PO2	SO2	BEB	HCO3	TCO2	HGB	FIO2
1090625	0146	7.312	40.8	87.5	95.4	-5.7	20.2	21.4		

DATE	TIME	PH	BEB	SCVO2	PO2	HCO3	HGB	PCO2	TCO2
1090629	0959	7.306	-4.4	86.5	58.5	22.0		45.0	23.3

特殊檢查

病理報告

其它

出院時情況

- 3.改門診治療

出院指示

-

藥名	劑量	劑量單位	用法	頻次	數量	數量單位	現況	天數	單位
Ultracet tab	1	TAB	PO	Q6H	12	TAB	TH	3	天
Pantoloc tab 40mg	1	TAB	PO	QDAC	3	TAB	TH	3	天
CefaDROxil-500 cap	1	CAP	PO	Q12H	6	CAP	TH	3	天

出院計劃

主治醫師 2222H 林俊余 / 住院醫師 9948L 吳柏寬

正式摘要**(**電子簽章：**000000195992918)**

W83 / W83

doctorRank:B
